# The effect of nutrient supplementation in the management of polycystic ovary syndrome-associated metabolic dysfunctions: A critical review

**DOI:** 10.4274/jtgga.2018.0077

**Published:** 2018-11-15

**Authors:** Elif Günalan, Aylin Yaba, Bayram Yılmaz

**Affiliations:** 1 Department of Physiology, Yeditepe University School of Medicine, İstanbul, Turkey; 2 Department of Histology and Embryology, Yeditepe University School of Medicine, İstanbul, Turkey

**Keywords:** Polycystic ovary syndrome, insulin resistance, hyperandrogenism, metabolic dysfunctions, dietary supplements

## Abstract

Polycystic ovary syndrome (PCOS) is complex heterogeneous disorder that has several aspects in terms of pathology such as metabolic, endocrine, reproductive, and psychological. However, the etiology of PCOS remains poorly understood. Several studies suggest that insulin resistance and hyperandrogenism play a central role in the progression of PCOS pathophysiology. Therefore, common treatment strategies of PCOS are based on lifestyle modification, which include exercise, diet, and nutrient supplementation therapy. Recent studies have recommended some nutrients such as vitamins, minerals, and vitamin-like nutrients for the therapy of PCOS because each has at least one functional property in PCOS-induced pathways. Therefore, it is claimed that the cause of PCOS could be vitamin or mineral deficiency. This review aims to provide a critical literature survey on nutritional supplementation for the treatment of PCOS-associated endocrine and metabolic dysfunctions and discuss the role of nutrients in the management of PCOS in view of the clinical trials and experimental studies.

## Introduction

The polycystic ovary syndrome (PCOS) is one of the most common endocrine diseases that affects 5 to 10% of women of adolescent and reproductive age (1,2). PCOS was first defined by Stein and Leventhal in 1935. The basic characteristic trait of PCOS is hyperandrogenism as a result of excessive androgen secretion or activity (3). However, hyperandrogenism is not the only diagnostic criteria for PCOS. According to the Rotterdam criteria, PCOS is defined by the existence of at least two of three criteria, which are hyperandrogenism, chronic anovulation, and polycystic ovaries on ultrasound findings (4). Later, the Androgen Excess and PCOS Society reported that there should be the presence of hyperandrogenism and ovarian dysfunction (anovulation and polycystic ovaries) for PCOS (5). Patients with PCOS have various symptoms including menstrual dysfunction, hyperinsulinemia, infertility, glucose intolerance, type 2 diabetes, hirsutism, obesity, acne, metabolic syndrome, increased risk for the development of cardiovascular diseases, endometrium cancer, anxiety, obstructive sleep apnea, and abnormalities of lipid profile (6,7).

Although there are extensive studies in the literature, the cause of PCOS remains unclear due to poorly understood interactions between genetic and environmental factors (8). Reproductive neuroendocrine defects, impaired ovarian steroidogenesis, insulin resistance (IR), and increased cortisol metabolism-related adrenal hyperandrogenism can be among the causes of PCOS (9,10,11). Recent studies suggest that IR contributes to both metabolic and reproductive disturbances. Therefore, IR has a central role in the pathogenesis of PCOS (12). Briefly, insulin is considered as a key hormone for hyperandrogenism in the PCOS pathophysiology via two different pathways: 1) insulin stimulates androgen production of theca cells with luteinizing hormone (LH) and elevated androgen production leads to hirsutism, acne, and anovulatory infertility. 2) Hyperandrogenism associated function of insulin is inhibition of sex hormone-binding globulin (SHBG) synthesis in the liver (13). SHBG is a plasma protein for androgen and estrogens and so decreased SHBG levels can lead to hyperandrogenism in PCOS. At the metabolic perspective, insulin plays a key role in regulating glucose metabolism, blocking of lipolysis, and activation of aminoacid transportation (14). Various nutrients have regulatory roles in the insulin signaling pathway and androgen synthesis.

Providing sufficient nutrients and energy for growth and reproduction depend on the definition of the optimal nutrient composition. It is clear that nutrition-associated signaling pathways play a central role in the regulation of ovarian follicle growth and ovulation rates (15). In particular, deficiencies of myo-inositol and vitamin D can lead to PCOS pathogenesis-related complications (16,17,18). Therefore, nutritional supplementation may contribute to overcome complications of PCOS such as immature oocyte, IR, hyperandrogenism, and oxidative stress. This review addresses current knowledge about the efficacy of nutrients in the treatment of PCOS in view of experimental studies and clinical studies.

## Vitamin Supplements

**Vitamin A** is a fat soluble vitamin also known as retinol. Vitamin A-derived metabolites such as retinoids, retinoic acid, and retinol contribute to antioxidant activity, steroid metabolism, oocyte nuclear maturation, and inhibition of cumulus cell apoptosis (19,20). It is known that retinoic acid synthesis-related genes are expressed differentially in theca interna cells isolated from patients with PCOS (21). To examine the effects of retinol and retinoids, derivatives of retinol were applied into theca interna cell culture obtained from PCOS and healthy women. All trans retinol-treated theca interna cells gave rise to increased dehydroepiandestrone levels and mRNA accumulation of cytochrome P450 17α hydrxylase (CYP17) involved in androgen production and retinol biosynthesis (22). Obesity and abnormal glucose metabolism are associated with elevated retinol-binding protein 4 (RBP4) levels in overweight women with PCOS (23). Another RBP4-based study reported the measurement of RBP4 expression in isolated subcutenous and omental adipose tissue from women with PCOS. The authors suggested that elevated 17β estrodiol could contribute to the altered gonadal and adrenal steroid profile via upregulation of the RBP4 gene (24).

**B group vitamins; **most studies focus on B6, B12, and folic acid in this group due to the increasing role of homocysteine (Hcy) in PCOS. In this mechanism, Hcy is an essential amino acid derived from dietary methionine and elevated total plasma Hcy levels lead to an increased risk for cardiovascular and reproductive symptoms in PCOS (25). In addition, other metabolic pathways required for growth of cell and tissue are closely associated with Hcy (26). Folic acid, vitamin B6, and vitamin B12 have significant roles in Hcy regulation. In the physiopathology of PCOS, a positive correlation has been reported between IR and elevated Hcy levels (27,28). Kaya et al. (29) demonstrated that IR, obesity, and increased Hcy levels were related to low serum insulin B12 concentrations in women with PCOS. In order to reduce elevated levels of serum Hcy, folic acid supplementation for three months produced effective results, especially in women without IR. However, a dose-dependent effect of folic acid supplementation is not known (30). Regular exercise has also been suggested to decrease plasma Hcy concentrations in the pathophysiology of PCOS. According to the study of Randeva et al. (31) regular exercise for a period of six months provides significantly lower plasma Hcy levels in young obese and overweight women with PCOS.

Many women with PCOS have to use insulin-sensitizing agents such as metformin for improving insulin sensitivity. Metformin inhibits the binding intrinsic factor-B_12_ complex and its receptor, and also serum vitamin B12 and folic acid levels decrease during metformin therapy (32). In addition, metformin increases Hcy levels; therefore it gives rise to the long-term risk of cardiovascular diseases in women with PCOS (33). The interaction between metformin and B group vitamins has been explained by two studies: the first report showed that daily administration of folic acid or B group vitamins could be effective in reducing elevated Hcy levels in women with PCOS in short-term metformin therapy. However, the authors also suggested that supplements of vitamins had no effects on androgen and lipid levels in the pathophysiology of PCOS (34). The second report showed that the use of metformin with folate supplementation for six months had beneficial effects on vascular endothelium. This treatment provides reduced Hcy levels, thus it can be effective in the management of the long-term complications of PCOS such as cardiovascular disorders (35)_._

**Inositol** and its metabolites are known as sugar alcohols and also belong to B complex vitamins. In addition, inositol has 9 stereoisomers such as myo-, cis-, allo-, epi-, muco-, neo-, scyllo-, D-chiro and L-chiro- forms (36). Inositol-derived metabolites have essential roles in insulin sensitivity as second messengers, lipid synthesis, signal transduction, oocyte maturation, oogenesis, cell morphogenesis, and cytoskeleton organization (37). According to randomized controlled studies involving inositol supplementation in women with PCOS, inositol provides improvement in almost all pathologic conditions in PCOS such as recovery of reproductive abnormalities, decreased androgen levels, and improved insulin levels (38).

Interestingly, combined treatment of inositol isomers such as myo-inositol (MI) and D-chiro inositol (DCI) should be applied at a certain ratio, which is known as the plasma physiologic ratio (MI/DCI: 40/1) (39). Otherwise, immature oocytes can appear, and the efficacy of inositol is decreased in the pathophysiology of PCOS (40). Some studies have claimed that these pathologic conditions may be accounted for by the ‘DCI paradox’ (41). Briefly, MI is found in the follicle-stimulating hormone (FSH) signaling mechanism and homeostasis of glucose uptake, and DCI is prompted to insulin-associated androgen synthesis. Epimerase plays a functional role in conversion of MI to CDI depending on insulin levels and also intake of inositol isomerase, except the physiologic ratio can lead to decreased MI and increased CDI levels. When hyperinsulinemia occurs in the pathogenesis of PCOS, elevated epimerase activity can lead to abnormalities in the FSH signaling pathway; therefore, immature oocytes and hyperandrogenism may develop (42).

Contributions of MI to treatment in women with PCOS are reviewed in Table 1 (43,44,45,46,47,48,49,50). According to the current literature, treatment of MI provides healing in hyperandrogenism and IR- associated parameters, and also improvement of the lipid profile.

**Vitamin D** is so essential vitamin for skeletal growth, regulation of serotonin synthesis, bone mineral density, dental health, lower extremity functions, and regulation of calcium (Ca) and phosphorus metabolism. In addition, previous studies reported that vitamin D might be a significant and independent predictor of IR (51). Vitamin D levels decrease in obese patients when compared with non-obese people owing to IR. Regarding to PCOS, a recently published review by Krul-Poel et al. (52) about the role of vitamin D in metabolic disturbances of PCOS confirmed an association between vitamin D and metabolic disturbances. Thereby, it was found that women with PCOS (who are obese) had significantly decreased 25-dehydroxy vitamin D levels (53). Moreover, a cross-sectional study reported that lower D vitamin was linked with IR as a result of the pathophysiology of PCOS (54).

Researchers focus on vitamin D supplementation for the treatment of women with PCOS to show an interaction between vitamin D deficiency and PCOS. A recent study about vitamin D replacement therapy in which vitamin D3 was administered for three weeks in 11 subjects with PCOS suggested some beneficial effects on IR, but no changes in androgen levels were observed (55). In addition, Kotsa et al. (56) used a vitamin D3 analogue (alphacalcidol) in order to determine the effect of vitamin D in the treatment of PCOS. Their findings showed an increased first phase of insulin secretion, decreased serum triglyceride (TG) levels, and increased serum high-density lipoprotein (HDL) cholesterol profile.

The molecular mechanism between vitamin D supplementation and improvement of PCOS is currently unknown. However, a recent report claimed that vitamin D3 replacement treatment  in women with PCOS improved some biochemical parameters by increasing in amount of soluble receptor for Advanced Glycosylated Ends (AGEs). Therefore, vitamin D3 inhibits inflammatory progress in the pathogenesis of PCOS. Moreover, vitamin D3 treatment plays a vital role in folliculogenesis due to decreasing elevated anti-mullerian hormone levels (57). Interestingly, Jafari-Sfidvajani et al. (58) demonstrated that vitamin D supplementation in women with PCOS caused no statistically significant differences in the androgen profile when combined with a low-calorie diet; however, an improvement in menstrual frequency was observed.

**Vitamin E** is a lipid-soluble vitamin and free radical scavenger that regulates the balance between antioxidant and oxidant systems (59). In addition, new evidence confirmed that vitamin E could improve endometrial thickness in women with unexplained infertility, and the effects were attributed to its anticoagulant and antioxidant effects (60). Moreover, cotreatment of coenzyme q10 and vitamin E for 8 weeks in patients with PCOS provided improvement in SHBG concentrations (61). Another study showed that vitamin E (400 IU) and omega-3 fatty acid (1000 mg) co-supplementation in women with PCOS for 12 weeks provided significant improvement in IR and androgen levels (62).

## Supplementation of Vitamin-like Nutrients in PCOS

**Alpha-Lipoic Acid (a-LA)** is a free radical scavenger, an essential cofactor in the citric acid cycle, and a regulatory agent of body weight (63,64). Interestingly, Masharani et al. (65) found that controlled release of α-LA administered to six non-diabetic women with PCOS was not related with elevation in plasma antioxidant potency or reduction in plasma oxidation metabolites. To investigate the role of α-LA and DCI (DCA) in the short-term management of PCOS, both metabolites were given to 46 women (26 women with PCOS and 20 female controls) for 180 days. They suggested that some reproductive characteristics improved including menstrual cycles, decreased number of ovarian cysts, and increased progesterone levels. At the metabolic perspective, IR significantly improved in the subjects wiht PCOS, and impaired lipid metabolism was significantly changed (66).

**Bioflavonoids** consist of polyphenolic compounds, which are found in plants. Flavonoids have antioxidant, antidiabetic, antiestrogenic, anti-inflammatory, and antiproliferative properties (67). Bioflavonoids consist of various metabolites, some of which provide improvement of the pathogenesis of PCOS at different levels. For instance, Oh et al. (68) analyzed six flavonoid classes (anthacyanides, flovan-3-oils, flavanones, flavones, flavonols and isoflavones) in terms of their contribution to the treatment of metabolic syndrome in PCOS pathophysiology. The authors suggested that only flavonol consumption was the most effective treatment of metabolic syndrome in PCOS when compared with the other groups (68). Romualdi et al. (69) showed that 36 mg/d soy isoflavone genistein treatment in women with PCOS for three months provided a significantly improved lipid profile. However, other characteristic traits of PCOS such as hyperinsulinemia, anthropometric measurements, hyperandrogenism, and reproductive abnormalities did not change significantly (69). On the contrary, in an experimental study on rats, Shah and Patel (70) reported improved ovarian and uterine morphologic appearences, increased LH levels, and significantly decreased insulin and testesterone in PCOS following quercetin treatment, a bioflavonoid with antioxidant activity. They considered that quarcetin was functional in phosphatidylinositole-3-kinase (PI3K) inhibition and therefore PI3K could be beneficial target for a novel therapy approach of PCOS (70).

**Carnitine** is a quaternary ammonium compound found in fatty acid metabolism, oxidative stress mechanisms, and glucose metabolism (71). According to a clinical study, non-obese women with PCOS have significantly decreased serum total L-carnitine levels when compared with healthy women (72). Fenkci et al. (72) considered that lower L-carnitine level could be linked with hyperandrogenism and IR. Consistently, some antidiabetic agents that are used for PCOS treatment are associated with carnitine metabolism. For instance, piaglitazone administration for 16 weeks in obese premenoupausal patients with PCOS led to increased fasting concentrations of free carnitine (73). Moreover, Dunning and Robker (74) claimed that L-carnitine influenced oocyte quality because L-carnitine provides transport of fatty acids and regulation of energy production, which have a central role in promoting oocyte maturation. Immature oocytes can be a source of metabolic and endocrine malfunctions in PCOS (75). A randomized clinical trial in clomiphene-resistant women with PCOS reported that using both clomiphene citrate and L-carnitine provided thicker endometrium, higher estradiol concentrations, higher pregnancy rates, and improved lipid profiles compared with clomiphene citrate treatment alone (76). Another study demonstrated that L-carnitin supplementation (250 mg per day) for 12 weeks had beneficial effects within mental health and oxidative stress parameters (77).

## Mineral Supplements

Mineral supplements are among the dietary supplements that are expexted to provide improvement of metabolic profile, mental health, ovulation, and menstrual cyclicity. Recent studies about PCOS focused on mineral supplementation in order to remove pathologic situations from PCOS. 

**Calcium** is an essential micronutrient and is involved in egg activity, oocyte maturation, progression of follicular development, and regulation of cell division in mammalian oocytes (78,79,80). Furthermore, Ca deficiency could be related to risk of obesity because the insulin signaling pathway is Ca dependent (81). Therefore, it is considered that abnormalities of Ca concentrations could be associated with IR and promoting PCOS pathologies. Biochemical studies have shown that decreased Ca levels are observed in obese women with PCOS when compared with healthy women. Ca homeostasis depends on vitamin D receptor (VDR), parathyroid hormone (PTH), and Ca-sensing receptor (CaSR). In addition, adiponectin concentration is strongly associated with Ca and vitamin D levels (82). To determine the role of the polymorphisms of Ca homeostasis-linked factors in initiating PCOS, VDR, PTH, CaSR, insulin receptor, and adiponectin genes were analyzed and compared with PCOS-associated biochemical parameters. Consequently, polymorphisms of VDR are related to increased LH and reduced SHBG levels and the gene variant of CaSR is linked to higher homeostatic model assessment-IR (HOMA-IR) and IR (83). Combined supplementation of vitamin D 100,000 IU/month, Ca 1000 mg/day, and metformin 1500 mg/day for 6 months in 100 infertile patients with PCOS resulted in significanly reduced body mass index (BMI). In addition, menstrual cyclicity, follicular maturation, and pregnancy rates were affected positively, but the alterations were not statistically significant (84).

**Chromium** is an essential mineral that has an essential role in carbohydrate and lipid metabolism. Chromium has been widely studied in the treatment of hyperglycemia, especially type 2 diabetes, because chromium deficiency leads to disorders in glucose homeostasis and IR (85). There is also evidence to confirm that women with PCOS showed decreased chromium levels, which was linked to IR (86). A pilot study suggested that with daily supplementation of 200 *µ*g chromium for three months, women with PCOS showed improved glucose tolerance, but it did not affect reproductive function and hormonal disturbances (87). Another study involving 64 women with PCOS showed that daily 200 *µ*g chromium supplementation for eight weeks caused significant decreases in serum insulin levels, HOMA-IR, HOMA-B, TGs, very-low-density lipoprotein (VLDL) cholesterol, and total cholesterol concentration. In addition, Jamilian and Asemi (88) showed that a significantly increased quantitative insulin sensitivity check index (QUICKI) score in women with PCOS compared with placebo. However, circulating LDL, HDL, cholesterol levels, and fasting plasma glucose levels were not altered in the treatment group (88). 

The effect of chromium within androgen level depends on the treatment amount and duration of chromium treatment. According to a double-blind, randomized clinical study, chromium picolinate (200 *µ*g/day) treatment in 46 patients with clomiphene citrate-resistant PCOS for 3 months gave rise to increased insulin sensitivity. However, there were no findings about a relationship between applied chromium and androgen levels (89). 

In contrast, Amr and Abdel-Rahim (90) administered high doses of chromium picolinate (1000 *µ*g/day) treatment to adolescent girls with PCOS for 6 months. At the end of the study, improvement of oligo/amenorrhea, decreased number of total follicles, lower free testesterone levels, and smaller ovarian volume were obtained in ultrasonographic views and biochemical analyses.

**Magnesium** is the one of the most predominant intracellular cations (91). Magnesium regulates ATP-generation, ATP-use, transphosphorylation reactions, DNA and RNA synthesis, insulin metabolism, ion homeostasis, membrane structure, cytoskeletal function, and cell growth (92). In addition, magnesium is associated with entry of Ca into the neuron because magnesium is a Ca antogonist and a voltage-dependent blocker of the N-methyl-D-aspartate channel (93,94). This property provides protection for neurons against cell death. Therefore, magnesium supplementation is used generally in neurologic disorders including depression-related diseases such as PCOS, as well hypertension, cardiovascular diseases, and diabetes (95,96). However, only a few studies have suggested a relationship between serum magnesium level and the pathogenesis of PCOS. Lower serum magnesium level and higher Ca/Mg ratios in women with PCOS due to IR have been reported. No significant correlation between Mg levels and steroid hormones was found (97). The effects of magnesium levels in PCOS pathology remains unclear.

**Selenium** is an effective essential element against oxidative stress and is required for the embryonic gonodal development and function of reproductive tissues (98). Biochemical studies have shown that women with PCOS have lower selenium level compared with controls. Coskun et al. (99) suggested that accumulation of free radicals was detected in PCOS women due to insufficient selenium level, which leads to increased androgen levels including LH and total testesterone (99). In this regard, selenium supplementation in the form of immunomodulatory drug (IMOD) was administered for 21 days to hyperandrogenism-induced PCOS female rats. IMOD reduced tumor necrosis factor-α production and increased antioxidant capacity (100).

Another aspect of selenium intake is related to glucose and fat metabolism because selenium possesses insulin-like activities (101,102). There were two clinical studies about the effect of selenium supplementation in women with PCOS in terms of IR. In the first study, 70 women with PCOS were randomly divided into two groups, one received 200 *µ*g per day selenium supplements (n=35) and the other placebo (n=35). After 8 weeks of intervention, they reported a reduction in serum insulin levels, HOMA-IR, HOMA-B, and increased QUICKI. Also, selenium intake showed decreased serum TGs and VLDL-C concentrations when compared with placebo (103). Another study included 200 *µ*g selenium supplementation (n=20) and placebo (n=20) per day for 8 weeks in 40 infertile women with PCOS. At the end of the study, the authors measured the insulin and lipid-related gene expression levels such as PPAR-γ, GLUT-1, and LDLR from lymphocytes in the subjects. The results showed that selenium supplementation could be a candidate for* in vitro* fertilization due to significantly increased expression levels of PPAR-γ and GLUT-1 and decreased expression levels of LDLR (102).

**Zinc** is an another essential trace element found in the metabolism of lipid, carbohydrates, and protein, which is responsible for the function of over 300 enzymes. It is a component of more than 200 enzymes (104). In particular, zinc ions play crucial roles in insulin metabolism including the synthesis, storage, secretion, conformational integrity, function, and action of insulin, and also zinc ions produce an insulin-like effect (105). For this reason, insufficiency of zinc gives rise to diabetes, obesity, glucose intolerance, lipidemia, hyperglycemia, and hypertriglyceridemia (106,107). Studies have shown that women with PCOS have lower zinc levels (108). It has been demonstrated that one of the reasons for IR in PCOS was related to decreased insulin-dependent tyrosine phosphorylation due to a post-receptor defect (109,110). Therefore, inadequate zinc levels could not stimulate insulin receptor tyrosine kinase in patients with PCOS. Zinc levels can play an important role in the development of IR in PCOS. Several studies suggested that zinc supplementation had therapeutic effects for the prevention of type 2 diabetes (111).

The pathology of PCOS involves risk of cardiovascular diseases in the long term due to altered lipid profiles including elevated trygliceride levels, decreased HDL levels, and increased LDL levels (112). It has been suggested that zinc deficiency in PCOS might be associated with abnormal lipid profiles. The effect of zinc supplementation in women with PCOS has been shown in recent clinical research (113). In this study, 50 mg/d of zinc as zinc sulphate or placebo was given to 60 women with PCOS for 8 weeks, as an adjunct to their pre-study oral estrogen-progestrone compound therapy. The results showed a significant reduction in levels of serum total cholesterol, LDL-C, TG, and TG/HDL-C ratio in the zinc group (113). Therefore, zinc supplementation can provide an effective adjunctive nutritional therapy with potential for improving lipid metabolism and IR in women with PCOS.

## Other Supplements

**Melatonin (MT) **is a neuroendocrine hormone secreted from the pineal gland. It plays a central role in the regulation of circadian rhythm. High concentrations of MT have been found in follicular fluid, which affects physiologic processes in the ovaries such as folliculogenesis, follicular atresia, ovulation, steroidogenesis in theca cells, and corpus luteum formation due to its powerful free radical scavenger activity (114,115,116). Moreover, Wei et al. (117) reported that supplementation of MT at a low concentration supports nuclear maturation of oocytes *in vitro.* Therefore, MT may provide improvement of oocyte quality and increase pregnancy rates (118). Concentration of MT in pre-ovulatory follicular fluid is lower in women with PCOS. Kim et al. (119) suggested that MT administration may be useful in *in vitro* fertilization strategy and improve clinical outcomes of PCOS.

**N-acetyl-L-cysteine (NAC)** is the acylated form of L-cysteine amino acid and also one of the precursors of glutathione, an antioxidant substance (120). Liu et al. (121) showed that NAC administration supported oocyte quality through an anti-aging effect on mouse oocytes. In addition, NAC regulates insulin receptor function in eryhtrocytes and supports insulin secretion from the pancreatic β cells (122). Fulghesu et al. (123) investigated the effects of NAC administration for 5-6 weeks on insulin-associated parameters in obese and lean women. They determined a significant decrease in testesterone and androgen levels. In addition, increased peripheral insulin sensitivity appeared in women with PCOS (123). Thus, both metformin and NAC have important effects on hyperandrogenism, hyperinsulinemia, and menstrual cyclicity in women with PCOS. Elnashar et al. (124) compared the effects of metformin and NAC on insulin and testesterone levels and ovulation success in women with clomiphene citrate-resistant PCOS. In fact, clomiphene citrate is used in the first-line treatment of PCOS as a stimulator of ovulation. However, resistance against clomiphene citrate in women with PCOS obstructs the possibility of pregnancy. As a consequence, it was suggested that metformin had more efficacy in ovulation rates (51.6%) and insulin sensitivity than NAC (124). Another clinical trial compared metformin use (500 mg three times daily) and NAC supplementation (600 mg three times daily) over a 24-week period. Both groups had equal efficacy in terms of decreased BMI and free testesterone levels, improved insulin sensitivity, menstrual cyclicity, and lower hirsutism scores. Moreover, metformin administration caused a decrease in total cholesterol levels and NAC supplementation led to reduction in both total cholesterol and LDL levels (125).

**Omega 3 Fatty Acids** are polyunsaturated fatty acids (PUFAs). α-linolenic acid, eicosapentaenoic acid (EPA), and docosahexaenoic acid (DHA) are the most commnly known members in this group. Each fatty acid has distinct metabolic and endocrine properties and PUFAs intake can be linked to reduced TG, whereas monounsaturated fatty acids (MUFAs) consumption leads to decreased testesterone level (126). Omega-3 fatty acids reduce oxidative stress, decrease hypertension, and improve lipid profiles and anti-inflamatory activity, and so they have potential role against cardiovascular disease risk (127,128). In recent years, omega-3 fatty acids have been considered as therapeutical agents for the treatment of PCOS. It has considered that the healing mechanism of omega-3 is associated with regulation of abnormal gene expression in the pathophysiology of PCOS. For instance, different doses (25-100 µg) of omega-3 EPA in granulosa cell culture resulted with higher insulin growth factor (IGF)-1 expression and lower cycloxogenase 2 (COX2) expression. It is clear that IGF-1 is an essential compound of follicular differentiation and COX-2 contributes to oocyte maturation (129).

The relationship between IR and omega-3 supplementation has been discussed by various researchers due to inconsistent findings. However, a meta-analysis about the effects of omega-3 in the IR-associated pathology of PCOS reported no association between intake of omega-3 and insulin sensitivity (130). Some clinical studies are briefly summarized in Table 2 (131,132,133,134,135,136).

**Probiotics** are living microbial dietary supplements found in dairy products and have synergism with the gut microbiota (137). Probiotics have beneficial effects in metabolism, especially under inflammatory conditions (138,139). According to recent studies, probiotic consumption improves fasting blood glucose and antioxidant status in patients with type 2 diabetes (140). In addition, Yadav et al. (141) showed that a probiotic-supplemented diet delayed the onset of glucose intolerence, hyperglycemia, hyperinsulinemia, and dyslipidemia in diabetic rats. Shoaei et al. (142) studied the effects of probiotic supplementation on pancreatic β cells and C-reactive protein (CRP) in patients with PCOS using multispecies probiotics for 8 weeks. The results of their study showed reduced fasting blood sugar and serum insulin levels in a crude model. Interestingly, CRP levels did not significantly change.

The etiology of PCOS has two pathologic conditions including a chronic state of inflammation and IR (143). Both conditions are associated with the dysbiosis of gut microbiota (DOGMA) theory. The background of DOGMA involves an imbalance in gut microbiota, i.e., increasing the transition of Gram-negative colonic bacteria into the systemic circulation. Therefore, a chronic inflammatory response occurs in the host. The inflammatory process affects insulin receptor function and PCOS-associated pathways such as androgen biosynthesis. Therefore, to overcome the pathophysiologic conditions of PCOS, probiotic supplements are recommended by some researchers (144,145). On this point, Guo et al. (146) performed fecal microbiota transplantation (FMT) and *lactobacillus* transplantation in rats with PCOS. At the end of the study, they reported that all rats in the FMT group had an improved estrous cycle and most of the lactobacillus-treated rats had decreased androgen biosynthesis (146).

**Figure 1:**
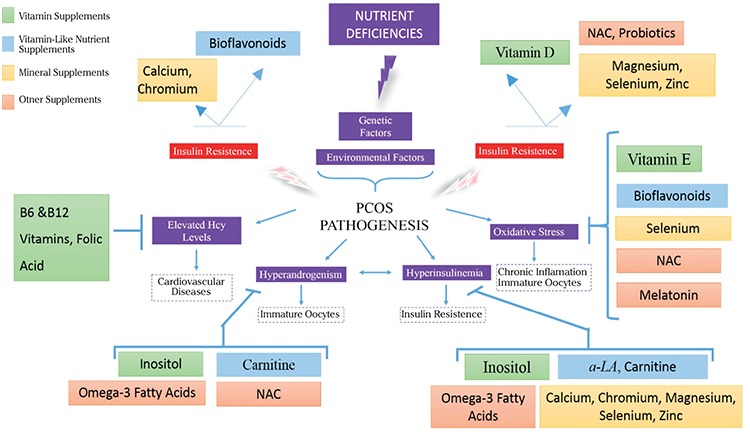
The effects of vitamins, minerals, vitamin-like substances and other supplements on the pathophysiology of PCOS *NAC: N-acetyl-L-cysteine; α-LA: α-linolenic acid; PCOS: Polycystic ovary syndrome*

The pathophysiology of PCOS is associated with various defects, including neuroendocrine defects, impaired ovarian steroidogenesis, IR, and increased cortisol metabolism-related adrenal hyperandrogenism. Although the triggering cause of PCOS is currently unknown, androgens and insulin are thought to be two key factors in its pathogenesis. Therefore, treatment of PCOS is required to overcome both hyperandrogenism and hyperinsulinemia. Nutrients act as cofactors in maintaining functions of insulin and androgen receptors. In this study, we focused on the efficacy of nutrient supplementation in management of PCOS because almost all vitamin and mineral deficiencies are seen in PCOS. In this process, published clinical and experimental studies that met specified criteria were extracted from PubMed, Web of Science, EmBASE, Google Scholar database from the last 25 years as accurately and precisely as possible. Articles were divided into treated nutrient groups; vitamins, minerals, vitamin-like substances, and other nutrients and each substance was evaluated in terms of treated dose, duration, and effectiveness in terms of their ability to prevent PCOS complications. In addition, we summarized how supplementation of different vitamins, minerals, and other supplements contribute to prevent complications of PCOS (Figure 1).

Figure 1 indicates that interactions between genetic factors and some nutrient deficiencies cause PCOS pathophysiology-related symptoms such as elevated Hcy levels, oxidative stress, hyperandrogenism, and hyperinsulinemia. In particular, deficiencies of vitamin D, bioflavonoids, Ca, chromium, NAC, probiotics, magnesium, zinc, and selenium are associated with IR. Therefore, the treatment of women with PCOS with these supplements provides improvement for hyperinsulinemia and increased insulin sensitivity. Inositol, vitamin A, carnitine, omega-3 fatty acids, and NAC supplements affect hyperandrogenism. Inositol and omega 3 supplementation in particular help the recovery of PCOS with regard to metabolic and reproductive parameters. Apart from that, vitamin B6, B12, and folic acid have beneficial effects in abnormal Hcy levels and also vitamin E, α-linolenic acid, bioflavonoids, selenium, NAC, and MT supplements help to remove the oxidative stress of PCOS. Nevertheless, the safe use and effectiveness of herbal medicine and nutrient supplements, except for inositol and omega-3 fatty acid, have not been clearly demonstrated and more studies are needed in these areas (147).

### Study Limitations

A limitation of our study was the huge number of related articles published: the doses, types, and combinations of supplemented nutrients are extremely different from each other, which depends on the investigated group, thus it makes the evaluation of the results difficult. Another limitation is the dose of nutrients used in the studies, as well as the insufficient diagnostic criteria used for PCOS. In addition, each woman with PCOS requires different supplementation depending on the signs and physiologic abnormalities. For instance, some patients have infertility due to PCOS, whereas others have endocrine and metabolic dysfunctions. However, most nutrient supplementation research focuses on the metabolic aspects of PCOS. Therefore, this review mostly focused on the therapeutic effects on the metabolic and endocrine dysfunctions instead of infertility, which is also a limiting factor in this study. Large, molecular scale up studies can be planned to illuminate the disrupted signaling pathways in PCOS. In this way, nutrients can be used effectively in the management of all aspects of PCOS via a molecular targeting strategy. 

In conclusion, vitamin or mineral supplements can exert beneficial effects on PCOS-related symptoms such as immature oocytes, hyperinsulinemia, hyperandrogenism, increased BMI, cardiovascular disorders, and mental and psychological problems.

## Figures and Tables

**Table 1:**
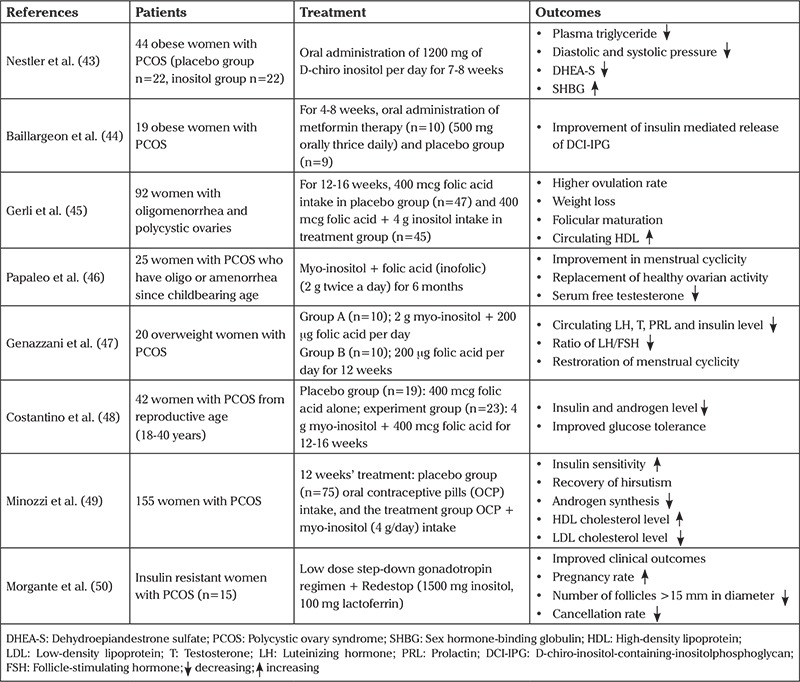
Effects of myo-inositol compounds in women with PCOS

**Table 2:**
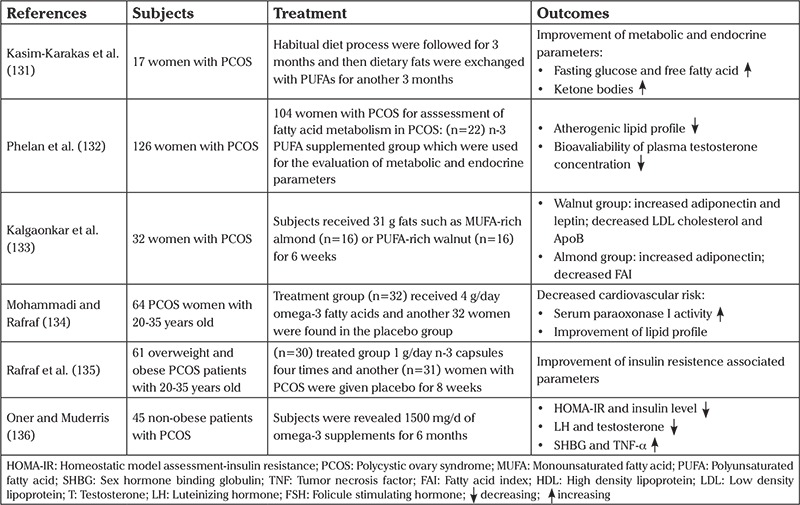
According to clinical studies, the role of omega-3 in the treatment of PCOS
